# Correction: Meneguzzo et al. Short-Term Effects of Forest Therapy on Mood States: A Pilot Study. *Int. J. Environ. Res. Public Health* 2021, *18*, 9509

**DOI:** 10.3390/ijerph19053034

**Published:** 2022-03-04

**Authors:** Francesco Meneguzzo, Lorenzo Albanese, Michele Antonelli, Rita Baraldi, Francesco Riccardo Becheri, Francesco Centritto, Davide Donelli, Franco Finelli, Fabio Firenzuoli, Giovanni Margheritini, Valentina Maggini, Sara Nardini, Marta Regina, Federica Zabini, Luisa Neri

**Affiliations:** 1 Institute of Bioeconomy, National Research Council, 10 Via Madonna del Piano, I-50019 Sesto Fiorentino, Italy; lorenzo.albanese@cnr.it; 2 Italian Alpine Club, 19 Via E. Petrella, I-20124 Milano, Italy; franco.finelli55@libero.it (F.F.); giomarghe@yahoo.com (G.M.); 3 Local Public Health Authority, AUSL- IRCCS, I-42122 Reggio Emilia, Italy; michele.antonelli@ausl.re.it (M.A.); davide.donelli@ausl.re.it (D.D.); 4 Institute of Bioeconomy, National Research Council, 101 Via Gobetti, I-40129 Bologna, Italy; rita.baraldi@cnr.it (R.B.); luisa.neri@ibe.cnr.it (L.N.); 5 Pian dei Termini Forest Therapy Station, 2311 Via Pratorsi, I-51028 San Marcello Piteglio, Italy; ricerca@terapiaforestale.it; 6 “Cesare Alfieri” Political Science School, University of Florence, 60 Via Vittorio Locchi, I-50141 Firenze, Italy; francescocentritto08@gmail.com; 7 CERFIT, Careggi University Hospital, I-50134 Florence, Italy; fabio.firenzuoli@unifi.it (F.F.); valentina.maggini@unifi.it (V.M.); 8 A.M.I.S.I. Italian Medical Association for the Study of Hypnosis, 28 Via Paisiello, I-20131 Milano, Italy; nardini_s@libero.it; 9 Mindfulness Association UK, Edinburgh EH9 1AR, UK; marta@martaregina.com

The authors wish to make the following corrections to this paper [1]: 

## Text Correction

There was an error in the original publication. Although the description of the control session FTSC, which was performed in an urban park, stood out in the original text and its difference in comparison with the others could be somehow guessed, the original text was not clear enough. Based on the short description of the method of conducting used in session FTSC, it is apparent that it was generically based on the program illustrated in Section 2.2, but substantially simplified. The statement to be corrected, on page 5 of the original paper, read as: “The control session FTSC was performed with professional guidance in an urban park downtown the city of Florence, Italy”.

A correction has been made to Section 2. Materials and Methods, 2.1. Forest Therapy Sessions: Basic Features and Measures, Page 5:

“However, the control session FTSC, which was performed in an urban park downtown the city of Florence, Italy, although including professional guidance, was carried out according to a method of conducting that was different and substantially simplified in comparison with the method illustrated in Section 2.2, in particular based on the immersion in nature with mindful use of the senses of sight, hearing, touch and smell.”

There was an error in the original publication. The average “rest” time was sometimes as long as 1 h, also due to the time required for filling the informed consent and POMS questionnaire, and listening to the speech. However, there was considerable variability from session to session, also related to logistic issues and several questions and requests for information issued by the participants. No one participant started a forest therapy session within half an hour after her/his arrival. The statement to be corrected, on page 7 of the original paper, read as: “After a rest period of one hour, introduced to relieve transport-related stress, every participant was presented with a reduced Profile of Mood States (POMS) questionnaire, which was completely anonymous and included information about gender, age, place of residence, job and habits such as smoking and sports”.

A correction has been made to Section 2. Materials and Methods, 2.1. Forest Therapy Sessions: Basic Features and Measures, Page 7:

“Overall, at least half an hour, and up to one hour, elapsed before participants started the forest therapy session, during which every participant was presented also with a reduced Profile of Mood States (POMS) questionnaire and listened to a short speech. Such time could have helped relieving transport-related stress. The POMS questionnaire was completely anonymous and included information about gender, age, place of residence, job and habits such as smoking and sports.”

There was an error in the original publication. Similar to the error performed on page 5 and referred to above, there was an unintentional mistake on page 8 of the original paper, as it remained from an earlier version of the article, where there was no such session FTSC. The statement to be corrected, on page 8 of the original paper, read as: “Except for session FTS1, a professional psychologist or psychotherapist took part in each of the other forest therapy sessions, according to a program named Forestfulness^®^”.

A correction has been made to Section 2. Materials and Methods, 2.2. The Method of Conducting the Forest Therapy Sessions, Page 8:

“Except for session FTS1, and for session FTSC that was carried out according to a different and simplified method of conducting referred to in Section 2.1, a professional psychologist or psychotherapist took part in each of the other forest therapy sessions, according to a program named Forestfulness^®^.”

There was an error in the original publication. The program Forestfulness^®^ did not apply to the control session FTSC. There was an unintentional mistake on page 9 of the original paper. The statement to be corrected, on page 9 of the original paper, read as: “During the structured forest therapy sessions (FTS2 to FTS7 and FTSC listed in Table 1)”.

A correction has been made to Section 2. Materials and Methods, 2.2. The Method of Conducting the Forest Therapy Sessions, Page 9:

“During the structured forest therapy sessions (FTS2 to FTS7 listed in Table 1)”.

There was an error in the original publication. Only professionally conducted forest therapy sessions in remote forest areas, thus excluding FTSC that was performed in an urban park, were carried out according to the program illustrated in Section 2.2. The statement to be corrected, on page 16 of the original paper, read as: “All professionally conducted forest therapy sessions followed the same program, thus the effect of different programs could not be investigated”.

A correction has been made to Section 4. Discussion, 4.3. Limitations and Preliminary Technical Guidelines, Page 16:

“All professionally conducted forest therapy sessions in remote forest areas followed the same program, thus the effect of different programs could not be investigated.”

There was an error in the original publication. The paper was not aimed at assessing the importance of any method of conducting. Therefore, the final statement in the second paragraph of Section 5. Conclusions does not affect any results or other conclusions of the article. The statement to be removed, on page 18 of the original paper, read as: “The importance of the method of conducting could not be safely assumed based on the available data”.

A correction has been made to Section 5. Conclusions, Page 18:

Removed statement: “The importance of the method of conducting could not be safely assumed based on the available data”.

The authors apologize for any inconvenience caused and state that the scientific conclusions are unaffected. The original publication has also been updated.
